# Extraction of total RNA from single-oocytes and single-cell mRNA sequencing of swine oocytes

**DOI:** 10.1186/s13104-018-3264-2

**Published:** 2018-02-27

**Authors:** Katelyn M. Kimble, Sarah E. Dickinson, Fernando H. Biase

**Affiliations:** Department of Animal Sciences, Auburn University, 559 Devall Dr, Auburn, AL 36839 USA

**Keywords:** Oocyte, Embryo, RNA sequencing, Single-cell analysis

## Abstract

**Objective:**

Analyses of single oocytes are essential for a fine dissection of molecular features governing developmental competence. We adapted the phenol–chloroform procedure for the purification of total RNA from single oocytes.

**Results:**

Key modifications include the use of Phasemaker™ tubes, a second chloroform wash of the aqueous phase, and the precipitation of the RNA with glyclogen in a 200 μl micro-centrifuge tube. Assessment of the RNA profile from single oocytes showed distinct peaks for 18S and 28S ribosomal subunits. This approach permitted the extraction of small RNAs from single oocytes, which was evident by the presence of 5S and 5.8S rRNAs and tRNAs around 122–123 nucleotides long. The amplification of polyadenylated RNA resulted in detectable DNA products ranging from ~ 500 to ~ 5000 nucleotides. We used the amplified DNA as template for single-cell mRNA-sequencing of five swine oocytes and quantified the expression levels of 9587 genes with complete coverage of transcripts over 10,000 nucleotides in length. The coverage was similar in all oocytes sequenced, demonstrating consistent high RNA quality across samples. We isolated total RNA from single oocytes and demonstrated that the quality was appropriate for single-cell mRNA-sequencing.

**Electronic supplementary material:**

The online version of this article (10.1186/s13104-018-3264-2) contains supplementary material, which is available to authorized users.

## Introduction

The abundance of specific ribonucleic acids (RNAs) stored in the female gamete, namely the oocyte, has a direct relationship with the acquisition of developmental competence [1–3]. With the recent advances of next generation sequencing, single-oocyte RNA sequencing data have been generated for mice [4–6], cattle [7], goats [8], and humans [9]. Analyses of single oocytes are essential for a fine dissection of molecular features governing developmental competence, however, technical challenges must be overcome for the generation of data from oocytes compatible with massive single-cell sequencing.

In mammals, the collection of oocytes and downstream preparation for single-cell RNA sequencing is dependent on human handling. The RNA from single oocytes can be obtained through extraction kits [7] or exposed by cellular lysis [10] and used in enzymatic assays. However, three critical problems emerge from current approaches. First, most extraction kits dedicated to limited amounts of RNA work by selecting polyadenylated (polyA+) RNAs and/or using columns to selectively eliminate cellular debris and small RNAs. Second, it is unclear how much of the RNA is lost when small volumes of eluent are used to recover limited amounts of RNA. Third, oocytes store massive amounts of RNAs, proteins, and lipids. By lysing the cells with detergents, cellular debris can reduce the efficiency of downstream assays such as reverse transcription and polymerase chain reaction [11]. Improving the methods for extraction of RNA from single oocytes is necessary for us to generate next-generation sequencing data from different RNA species [i.e.: micro RNA, or mRNA (messenger RNA)] from single oocytes.

Motivated by the abovementioned limitations, in this report, we describe modifications to the phenol–chloroform [12] procedure that made the purification of total RNA from single oocytes possible. We demonstrate that the total RNA is suitable for the preparation of libraries for single cell mRNA. The approach we report can be applied to extract total RNA from single oocytes from any mammalian species.

## Main text

### Materials and methods

**Fig. 1:**
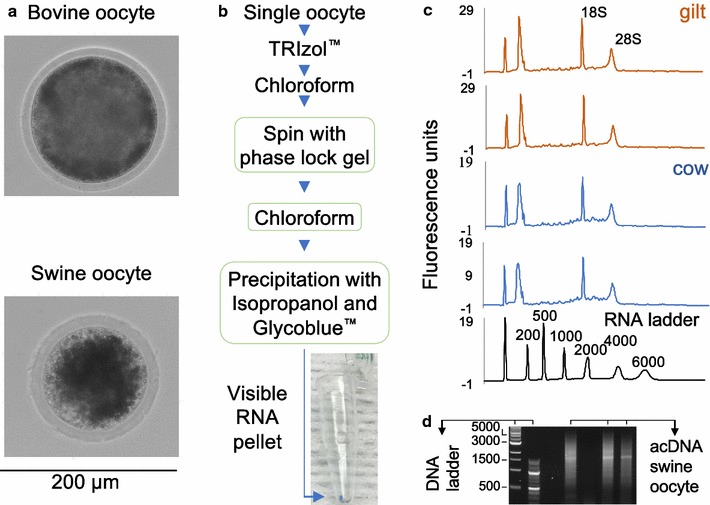
Total RNA extraction from single oocytes. **a** Representative images of bovine and swine oocytes. The scale bar is used for both images (**b**) Diagram of the RNA extraction flow. The green boxes indicate key adaptations to the standard TRIzol™ protocol. **c** Representative Bioanalyzer electropherograms of the total RNA extracted from single oocytes, and a ladder for reference. **d** Representative full-length cDNA amplification of swine oocytes

No live animals were handled for this study. Swine (*Sus scrofa*) and bovine (*Bos taurus*) ovaries were obtained from slaughterhouses and transported to the laboratory in saline solution for the manual aspiration of follicles and collection of cumulus-oocyte complexes. Single germinal vesicle oocytes were manually denuded of cumulus cells (Fig. 1a) and deposited into 5 µl of 1× phosphate-buffered saline (AMRESCO), supplemented with Ribonuclease inhibitor (0.5 U/μl) (AMRESCO), snap frozen in liquid nitrogen, and stored at − 80 °C. For the total RNA extraction from single oocytes (Fig. 1b), the incubation times and centrifugation steps were performed as recommended by the TRIzol™ (ThermoFisher) and Phasemaker™ (ThermoFisher) protocols. We thawed the oocyte by adding 150 μl of TRIzol™ followed by 30 μl of chloroform. The mixture was transferred to a Phasemaker™ tube for centrifugation at 12,000×*g* for 5 min at 4 °C. We then mixed the aqueous solution with 20 μl of chloroform followed by a second centrifugation at the same parameters. The aqueous solution was collected and mixed with 1 μl of Glycoblue™ (ThermoFisher) and 150 μl of isopropanol in a 200 μl tube, then subjected to a centrifugation at 12,000×*g* for 10 min at 4 °C. The RNA pellet was then washed twice with 75% ethanol followed by centrifugation at 7500×*g* for 5 min at 4 °C. The pellet was air dried and eluted in 1 μl of nuclease free water to profile the RNA length distribution in an Agilent 2100 Bioanalyzer (Agilent) following the manufacturer’s protocol.

To generate RNA-sequencing data from five swine oocytes, we eluted the RNA pellet in 4 μl of a solution containing oligo (dT) (Promega) and deoxynucleotide triphosphates (Promega) to assay full-length polyA+ RNA amplification following the SMART-seq2 procedure [10]. Following cleanup and quantification of the amplified complementary deoxyribonucleic acid (cDNA) on a Qubit 3.0 fluorometer (Thermo Fisher), 1 ng was used as template for next generation sequencing library preparation using Nextera XT DNA library Prep Kit (Illumina, Inc) [10]. The libraries were pooled and assayed on a HiSeq2500 instrument (Illumina, Inc) to generate pair-end reads.

The sequences were aligned to swine cDNAs obtained from Ensembl (Sscrofa11.1, release 90) using Bowtie2 [13] with the “–very-sensitive” option. Analyses to characterize the data properties were conducted in R software [14] (Additional file 1).

### Results and discussion

We assessed the distribution of total RNA from single bovine (N = 25) and swine (N = 22) oocytes (Fig. 1c). We observed distinct peaks for 18S ($\bar{x}_{peak} = 1740$ nucleotides (nt), bovine oocytes; $\bar{x}_{peak} = 1749$ nt, swine oocytes) and 28S ($\bar{x}_{peak} = 3481$ nt, bovine oocytes; $\bar{x}_{peak} = 4523$ nt, swine oocytes) ribosomal subunits. Additionally, we recorded RNA integrity number values between 5.1 and 7.3.

This procedure allowed us to profile small RNAs of single oocytes. The signal for small RNAs [5S, 5.8S, transfer RNAs (tRNAs)] is commonly observed in preparations using the phenol–chloroform without further column purification [15]. The peaks were averaged on 123 and 122 nt in the bovine and swine oocytes, respectively. The observations were consistent with the RNA length of the 5S ribosomal subunit [16].

**Fig. 2:**
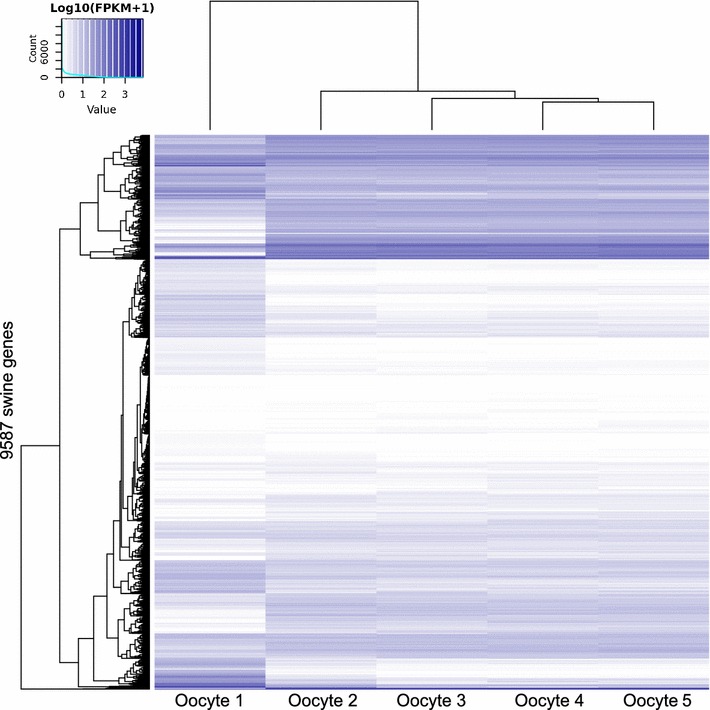
Heatmap of the genes expressed in single oocytes

Amplification of polyA+ RNA via polymerase chain reaction resulted in amplified cDNA (acDNA) products detected between ~ 500 and ~ 5000 nucleotides (Fig. 1d). The acDNA was used as input for library preparation and sequencing. We generated RNA-sequencing data from five swine single-oocytes averaging 9,680,369 pair-end reads. The alignment of the reads to the swine cDNA sequences resulted in of 9587 Ensembl genes detected in all five oocytes at with fragments per kilobase of transcript per million reads mapped > 0.3 (Fig. 2, see Additional file 2 for the list of genes).

**Fig. 3:**
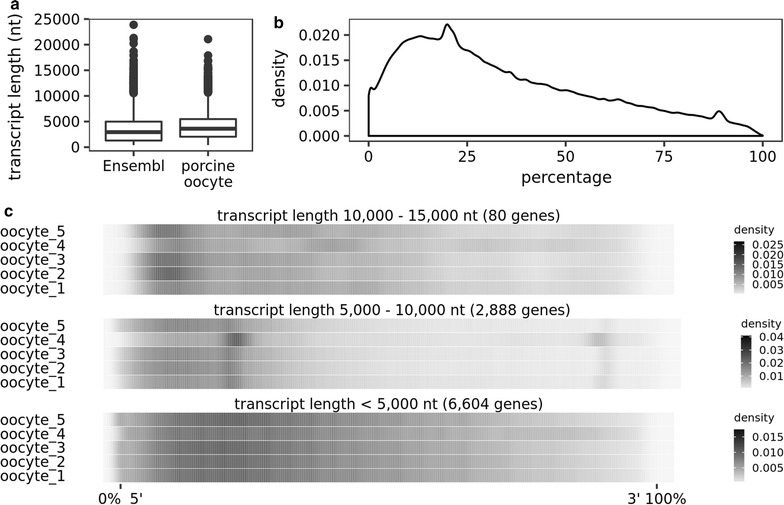
Single-cell mRNA sequencing of swine oocytes. **a** Length distribution of the transcripts sequenced in nucleotide units. The boxes represent the 25th, 50th, and 75th quantiles of the distribution. The lower error bar represents the lowest value > (− 1.5 × interquartile range) + 25th quantile. The upper error bar represents the greatest value < (1.5 × interquartile range) + 75th quantile. **b** Overall transcript coverage. **c** Transcript coverage for each oocyte in three different ranges of transcript length

The RNA-sequencing data revealed the quantification of short and long transcripts in single swine oocytes with a distribution similar to the observed in the Ensembl transcriptome database (Fig. 3a) with approximately 75% of the transcripts composed of ≤ 5000 nucleotides. Overall, there was a skewed distribution of reads towards 5′ end of the cDNA (Fig. 3b), which was probably caused by the tagmentation step of library preparation [17]. This tendency, however, was more prominent on transcripts whose length ranged between 5000 and 10,000 nucleotides, while, transcripts with less than 5000 nucleotides presented a more homogeneous read distribution across the transcripts (Fig. 3c). Most importantly, the overall distribution of read coverage on the transcripts was reproducible across five oocytes sequenced (Fig. 3c).

We adapted the phenol–chloroform protocol to extract total RNA from single oocytes. Our adaptations to the phenol–chloroform protocol permit non-selective extraction of total RNA from single oocytes. The extracted RNA is compatible with downstream enzymatic assays required for the generation of single-cell mRNA sequencing data. The transcripts of 9587 swine genes were sequenced from single oocytes with nearly full coverage.

## Limitations

Due to the limited number of cells analyzed, this study captured a snapshot of the complexity existent in bovine and swine oocytes.

## Additional files

**Additional file 1.** Extraction of total RNA from single-oocytes and single-cell mRNA sequencing of swine oocytes. This file contains the code used to generate Figs. 2 and 3.

**Additional file 2.** Genes expressed in single porcine oocytes. This file contains the genes expressed in single porcine oocytes and the corresponding annotation.

## Abbreviations

RNA: ribonucleic acid
rRNA: ribosomal ribonucleic acid
tRNA: transfer ribonucleic acid
DNA: deoxyribonucleic acid
mRNA: messenger ribonucleic acid
polyA+: polyadenylated
cDNA: complementary DNA
acDNA: amplified cDNA
nt: nucleotide
U: units
μl: microliter
