# Supplementary material for: Extraction of total RNA from single-oocytes and single-cell mRNA sequencing of swine oocytes
Source: BMC Res Notes. 2018 Feb 27;11:155. doi: 10.1186/s13104-018-3264-2 (PMC5828076; doi:10.1186/s13104-018-3264-2)
Supplement: Supplementary file 1 — Additional file 1. Extraction of total RNA from single-oocytes and single-cell mRNA sequencing of swine oocytes. This file contains the code used to generate Figs. 2 and 3. [file 13104_2018_3264_MOESM1_ESM.html]

Extraction of total RNA from single-oocytes and single-cell mRNA sequencing of swine oocytes


# Extraction of total RNA from single-oocytes and single-cell mRNA sequencing of swine oocytes

#### *Katelyn M. Kimble, Sarah E. Dickinson, Fernando H. Biase*

#### *January/2018*

Overview

This code accompanies the paper Kimble et al. (2018) Extraction of total RNA from single-oocytes and full-length single-cell mRNA sequencing of swine oocytes, as Supplementary file 1. Code created by Fernando Biase. Please, direct questions to *fbiase* at *auburn* dot *edu*.  
An updated version of this code may be available on www.biaselaboratory.com .

Code

Load the necessary libraries.

```
library(knitr)
library(Rsamtools)
library(stringr)
library(gplots)
library(ggplot2)
library(ggpubr)
library(scales)
library(kableExtra)
library(biomaRt)
library(RColorBrewer)
```

```
#function for collapsing the list of lists into a single list
#as per the Rsamtools vignette
.unlist <- function (x){
   ## do.call(c, ...) coerces factor to integer, which is undesired
   x1 <- x[[1L]]
   if (is.factor(x1)){
      structure(unlist(x), class = "factor", levels = levels(x1))
   } else {
      do.call(c, x)
   }
}
```

Load the annotations from Ensembl.

```
mart  = useMart("ensembl", dataset = "sscrofa_gene_ensembl",host="www.ensembl.org")
annotation.genelength.biomart<-getBM(attributes = c("ensembl_gene_id","ensembl_transcript_id", "transcript_length","gene_biotype"), mart = mart)
annotation.genelength.biomart<-annotation.genelength.biomart[annotation.genelength.biomart$gene_biotype =="protein_coding",]
annotation.genelength.biomart<-annotation.genelength.biomart[order(annotation.genelength.biomart$ensembl_gene_id, -annotation.genelength.biomart$transcript_length),]
#head(annotation.genelength.biomart[duplicated(annotation.genelength.biomart$ensembl_gene_id),])
annotation.genelength.biomart<-annotation.genelength.biomart[annotation.genelength.biomart$transcript_length > 400,]
annotation.genelength.biomart.a<-annotation.genelength.biomart[!duplicated(annotation.genelength.biomart$ensembl_gene_id),]
```

```
annotation.gene.biomart<-getBM(attributes = c("ensembl_gene_id", "hgnc_symbol","external_gene_name", "description","gene_biotype"), mart = mart)
annotation.gene.biomart<-annotation.gene.biomart[annotation.gene.biomart$gene_biotype =="protein_coding",]
```

Load bam files and generate the objects with data.

```
data_fpkm     <- list()
data_coverage <- list()

bamInPath<-"/home/fernando/oocyte_cumulus_pig/alignment_oocyte_cdna"

#read in BAM file
i<-1

for (sample in  c('SL269995','SL269992','SL270021','SL270003','SL269998')){
bam <- scanBam(paste(bamInPath,sample,"Aligned.out.merged.sorted.bam",sep="/"))
#store names of BAM fields
bam_field <- names(bam[[1]])
#go through each BAM field and unlist
list <- lapply(bam_field, function(y) .unlist(lapply(bam, "[[", y)))
#store as data frame
bam_df <- do.call("DataFrame", list)
names(bam_df) <- bam_field
bam_df<-as.data.frame(bam_df[,c(3,5,10)])
bam_df$transcript<-str_split_fixed(bam_df$rname, "\\.",2)[,1]
bam_df<-bam_df[,c(4,2,3)]

total_fragments_aligned<-dim(bam_df)[1]

bam_df_not_duplicated<-bam_df[bam_df$transcript %in% annotation.genelength.biomart.a$ensembl_transcript_id,]
table_transcript_count<-data.frame(table(bam_df_not_duplicated$transcript))
table_transcript_count<-merge(table_transcript_count, annotation.genelength.biomart.a, by.x="Var1", by.y="ensembl_transcript_id", all=FALSE)
table_transcript_count$fpkm<-( ( (table_transcript_count$Freq) / ((table_transcript_count$transcript_length/1000) * (total_fragments_aligned/1000000)) ) )
table_transcript_count<-table_transcript_count[,c(3,6)]
colnames(table_transcript_count)<-c("ensembl_gene_id", paste(sample))

data_fpkm[[i]]<-table_transcript_count


bam_df1<-bam_df[,c(1,2)]
bam_df2<-bam_df[,c(1,3)]
colnames(bam_df1)<-c("transcript","position")
colnames(bam_df2)<-c("transcript","position")
bam_df3<-rbind(bam_df1,bam_df2)
bam_df3<-bam_df3[!is.na(bam_df3$position),]

data_coverage[[i]]<-bam_df3

i<- i+1

rm(bam,bam_df,bam_df1,bam_df2,bam_df3,bam_df_not_duplicated, list)
}
```

Create the object that contains the expression data (FPKM) for the genes detected in all oocytes. The code used for saving the file is commented (#) and the table is printed below.

```
data_fpkm_a<-Reduce(function(x, y) merge(x, y, all=TRUE), data_fpkm)
rownames(data_fpkm_a)<-data_fpkm_a$ensembl_gene_id
data_fpkm_a<-data_fpkm_a[, c(2:dim(data_fpkm_a)[2])]
data_fpkm_b<-data_fpkm_a[rowSums(data_fpkm_a > 0.3) >=5,]
data_fpkm_b<-data_fpkm_b[complete.cases(data_fpkm_b),]
data_fpkm_b_annotated<-merge(data_fpkm_b, annotation.gene.biomart, by.x= "row.names", by.y="ensembl_gene_id", all.x=TRUE, all.y=FALSE)
colnames(data_fpkm_b_annotated)<-c("Ensembl ID","oocyte 1","oocyte 2","oocyte 3","oocyte 4","oocyte 5","hgnc_symbol","external_gene_name","description","gene_biotype")
```

Supplementary file 2.

```
kable(head(data_fpkm_b_annotated), format = "html") %>% kable_styling(bootstrap_options = c("striped", "hover", "condensed"),full_width =TRUE,font_size = 10)
```

| Ensembl ID | oocyte 1 | oocyte 2 | oocyte 3 | oocyte 4 | oocyte 5 | hgnc\_symbol | external\_gene\_name | description | gene\_biotype |
| --- | --- | --- | --- | --- | --- | --- | --- | --- | --- |
| ENSSSCG00000000002 | 3.054407 | 1.673888 | 1.5659515 | 1.370527 | 1.782784 | GTSE1 | GTSE1 | G2 and S-phase expressed 1 [Source:HGNC Symbol;Acc:HGNC:13698] | protein\_coding |
| ENSSSCG00000000005 | 3.272916 | 8.486265 | 3.1585472 | 4.837654 | 2.376952 | CDPF1 | CDPF1 | cysteine rich DPF motif domain containing 1 [Source:HGNC Symbol;Acc:HGNC:33710] | protein\_coding |
| ENSSSCG00000000006 | 17.612150 | 17.479475 | 18.5034100 | 4.667341 | 18.522566 |  | PPARA | Sus scrofa peroxisome proliferator activated receptor alpha (PPARA), mRNA. [Source:RefSeq mRNA;Acc:NM\_001044526] | protein\_coding |
| ENSSSCG00000000007 | 8.155091 | 11.088408 | 10.7312353 | 12.258417 | 8.616283 | TRMU | TRMU | tRNA 5-methylaminomethyl-2-thiouridylate methyltransferase [Source:HGNC Symbol;Acc:HGNC:25481] | protein\_coding |
| ENSSSCG00000000010 | 2.036271 | 1.823013 | 3.5314462 | 6.023551 | 1.844337 | FBLN1 | FBLN1 | fibulin 1 [Source:HGNC Symbol;Acc:HGNC:3600] | protein\_coding |
| ENSSSCG00000000014 | 1.089129 | 0.435553 | 0.7445085 | 1.954791 | 1.228299 | FAM118A | FAM118A | family with sequence similarity 118 member A [Source:HGNC Symbol;Acc:HGNC:1313] | protein\_coding |

```
data_fpkm_a[is.na(data_fpkm_a)]<-0
heatmap.2(log10(as.matrix(data_fpkm_a+1)),
        labRow=FALSE,
        labCol=FALSE,
        scale='none',
        trace='none',
        keysize=1,
        key.title='Log10(FPKM+1)',
        col=colorRampPalette(c("white", "blue4"))(n = 256),
        margins=c(0,0),
        key.par = list(cex=0.55))
```

```
data_coverage_a<-do.call("rbind", data_coverage)
data_coverage_a$sample<-c(rep(c("oocyte_1","oocyte_2","oocyte_3","oocyte_4","oocyte_5"), 
                              c(dim(data_coverage[[1]])[1], dim(data_coverage[[2]])[1],dim(data_coverage[[3]])[1],dim(data_coverage[[4]])[1],dim(data_coverage[[5]])[1]  )) )
data_coverage_a<-merge(data_coverage_a, annotation.genelength.biomart.a, by.x="transcript", by.y="ensembl_transcript_id", all=FALSE)
data_coverage_a<-data_coverage_a[data_coverage_a$ensembl_gene_id %in% rownames(data_fpkm_b),]
data_coverage_a$percentage<-data_coverage_a$position/data_coverage_a$transcript_length * 100
data_coverage_a<-data_coverage_a[data_coverage_a$percentage >0 & data_coverage_a$percentage < 100 ,]
```

Code to generate the individual images observed on Figure 2 on the paper.

```
font_1<-8
font_2<-6

data_fpkm_c<-merge(data_fpkm_b, annotation.genelength.biomart.a, by.x='row.names', by.y= 'ensembl_gene_id', all.x=TRUE, all.y=FALSE)
data_fpkm_c<-data_fpkm_c[,c(7,8)]
data_fpkm_c$sample<-"oocyte"
data_fpkm_c<-rbind(data_fpkm_c, data.frame('ensembl_transcript_id'=annotation.genelength.biomart.a$ensembl_gene_id, 'transcript_length'=annotation.genelength.biomart.a$transcript_length, sample='ensembl'))

plot1<-ggplot(data_fpkm_c, aes(x = sample, y = transcript_length)) +
       geom_boxplot() +
       scale_y_continuous(name = "transcript length (nt)")+
       scale_x_discrete(name = NULL, labels=c('Ensembl', 'porcine\noocyte')) +
       theme_bw()+
       theme(
         panel.background = element_blank(),
         plot.background = element_blank(), 
         panel.grid =element_blank(),
         axis.text  =element_text(colour="black", size = font_1),
         axis.title.y =element_text(colour="black", size = font_1, vjust=0, hjust=0.5)
            )

plot2<-ggplot(data_coverage_a, aes(x = percentage)) +
       geom_density()+
       theme_bw()+
       theme(
         panel.background = element_blank(),
         plot.background = element_blank(), 
         panel.grid =element_blank(),
         axis.text  =element_text(colour="black", size = font_1),
         axis.title =element_text(colour="black", size = font_1)
            )


data_coverage_b<-data_coverage_a[data_coverage_a$transcript_length >10000 & data_coverage_a$transcript_length<15000,]
data_coverage_c<-data.frame(
  'x_axis' = c( rep(density(data_coverage_b[data_coverage_b$sample=="oocyte_1",7])$x , 5)),
  'density'= c( density(data_coverage_b[data_coverage_b$sample=="oocyte_1",7])$y, density(data_coverage_b[data_coverage_b$sample=="oocyte_2",7])$y, 
                density(data_coverage_b[data_coverage_b$sample=="oocyte_3",7])$y, density(data_coverage_b[data_coverage_b$sample=="oocyte_4",7])$y,
                density(data_coverage_b[data_coverage_b$sample=="oocyte_5",7])$y),
    
  'sample' = c(rep(c("oocyte_1","oocyte_2","oocyte_3","oocyte_4","oocyte_5"), 
                   c( 
                     length(density(data_coverage_b[data_coverage_b$sample=="oocyte_1",7])$y), length(density(data_coverage_b[data_coverage_b$sample=="oocyte_2",7])$y), 
                     length(density(data_coverage_b[data_coverage_b$sample=="oocyte_3",7])$y), length(density(data_coverage_b[data_coverage_b$sample=="oocyte_4",7])$y),
                     length(density(data_coverage_b[data_coverage_b$sample=="oocyte_5",7])$y))
                     ) 
               ))

plot3<-ggplot(data_coverage_c, aes(x_axis, as.factor(sample))) + 
       geom_tile(aes(fill = density),colour = "white")+
       scale_fill_gradient(low = "#F0F0F0",high = "#000000")+
       ggtitle("transcript length 10,000 - 15,000 nt (80 genes)") +
       theme(
        panel.background = element_blank(),
        plot.background = element_blank(), 
        panel.grid =element_blank(),
       plot.title   =element_text(size=font_1, color="black",vjust = 0.4,hjust = 0.5, margin=margin(t =0, r = 0, b = 0, l = 0, unit = "pt")),
       axis.text.y  =element_text(size=font_1, color="black"),
       axis.text.x  =element_blank(),
       axis.title   =element_blank(),
       axis.ticks   =element_blank(),
       axis.line    =element_blank(),
       legend.title =element_text(size=font_2),
       legend.text  =element_text(size=font_2),
       plot.margin  =margin(t = 0, r = 0, b = 0, l = 0, unit = "pt"),
       legend.key.height=unit(0.2, "cm"),
       legend.key.width=unit(0.2, "cm")
       )


data_coverage_b<-data_coverage_a[data_coverage_a$transcript_length >5000 & data_coverage_a$transcript_length<10000,]
data_coverage_c<-data.frame(
  'x_axis' = c( rep(density(data_coverage_b[data_coverage_b$sample=="oocyte_1",7])$x , 5)),
  'density'= c( density(data_coverage_b[data_coverage_b$sample=="oocyte_1",7])$y, density(data_coverage_b[data_coverage_b$sample=="oocyte_2",7])$y, 
                density(data_coverage_b[data_coverage_b$sample=="oocyte_3",7])$y, density(data_coverage_b[data_coverage_b$sample=="oocyte_4",7])$y,
                density(data_coverage_b[data_coverage_b$sample=="oocyte_5",7])$y),
    
  'sample' = c(rep(c("oocyte_1","oocyte_2","oocyte_3","oocyte_4","oocyte_5"), 
                   c( 
                     length(density(data_coverage_b[data_coverage_b$sample=="oocyte_1",7])$y), length(density(data_coverage_b[data_coverage_b$sample=="oocyte_2",7])$y), 
                     length(density(data_coverage_b[data_coverage_b$sample=="oocyte_3",7])$y), length(density(data_coverage_b[data_coverage_b$sample=="oocyte_4",7])$y),
                     length(density(data_coverage_b[data_coverage_b$sample=="oocyte_5",7])$y))
                     ) 
               ))

plot4<-ggplot(data_coverage_c, aes(x_axis, as.factor(sample))) + 
       geom_tile(aes(fill = density),colour = "white")+
       scale_fill_gradient(low = "#F0F0F0",high = "#000000")+
       ggtitle("transcript length 5,000 - 10,000 nt (2,888 genes)") +
       theme(
       panel.background = element_blank(),
       plot.background = element_blank(), 
       panel.grid   =element_blank(),
       plot.title   =element_text(size=font_1, color="black",vjust = 0.4,hjust = 0.5, margin=margin(t =0, r = 0, b = 0, l = 0, unit = "pt")),
       axis.text.y  =element_text(size=font_1, color="black"),
       axis.text.x  =element_blank(),
       axis.title   =element_blank(),
       axis.ticks   =element_blank(),
       axis.line    =element_blank(),
       legend.title =element_text(size=font_2),
       legend.text  =element_text(size=font_2),
       plot.margin  =margin(t = 0, r = 0, b = 0, l = 0, unit = "pt"),
       legend.key.height=unit(0.2, "cm"),
       legend.key.width=unit(0.2, "cm")
       )

data_coverage_b<-data_coverage_a[ data_coverage_a$transcript_length<5000,]
data_coverage_c<-data.frame(
  'x_axis' = c( rep(density(data_coverage_b[data_coverage_b$sample=="oocyte_1",7])$x , 5)),
  'density'= c( density(data_coverage_b[data_coverage_b$sample=="oocyte_1",7])$y, density(data_coverage_b[data_coverage_b$sample=="oocyte_2",7])$y, 
                density(data_coverage_b[data_coverage_b$sample=="oocyte_3",7])$y, density(data_coverage_b[data_coverage_b$sample=="oocyte_4",7])$y,
                density(data_coverage_b[data_coverage_b$sample=="oocyte_5",7])$y),
    
  'sample' = c(rep(c("oocyte_1","oocyte_2","oocyte_3","oocyte_4","oocyte_5"), 
                   c( 
                     length(density(data_coverage_b[data_coverage_b$sample=="oocyte_1",7])$y), length(density(data_coverage_b[data_coverage_b$sample=="oocyte_2",7])$y), 
                     length(density(data_coverage_b[data_coverage_b$sample=="oocyte_3",7])$y), length(density(data_coverage_b[data_coverage_b$sample=="oocyte_4",7])$y),
                     length(density(data_coverage_b[data_coverage_b$sample=="oocyte_5",7])$y))
                     ) 
               ))

plot5<-ggplot(data_coverage_c, aes(x_axis, as.factor(sample))) + 
       geom_tile(aes(fill = density),colour = "white")+
       scale_fill_gradient(low = "#F0F0F0",high = "#000000")+
       ggtitle("transcript length < 5,000 nt (6,604 genes)") +
       scale_x_continuous(breaks = c(0,100), labels=c("0%  5'", "3' 100%"))+
       theme(
       panel.background = element_blank(),
       plot.background  = element_blank(), 
       panel.grid   =element_blank(),
       plot.title   =element_text(size=font_1, color="black",vjust = 0.4,hjust = 0.5, margin=margin(t =0, r = 0, b = 0, l = 0, unit = "pt")),
       axis.text.y  =element_text(size=font_1, color="black"),
       axis.text.x  =element_text(size=font_1, color="black"),
       axis.title   =element_blank(),
       axis.ticks.y =element_blank(),
       axis.line    =element_blank(),
       legend.title =element_text(size=font_2),
       legend.text  =element_text(size=font_2),
       plot.margin  =margin(t = 0, r = 0, b = 0, l = 0, unit = "pt"),
       legend.key.height=unit(0.2, "cm"),
       legend.key.width=unit(0.2, "cm")
       )
```

Next, we compiled individual plots to compose the panel observed on the paper. The image may seem distorted on the html file, but is not if saved as a png file.

```
ggarrange( ggarrange(plot1, plot2,labels = c('a', 'b'), ncol=2, nrow=1,  widths = c(2,4),hjust=c(0,0), font.label=list(size = 9, color = "black", face = "plain")),
           ggarrange(plot3, labels = c('c'), ncol=1, nrow=1,hjust=0, vjust=1,font.label=list(size = 9, color = "black", face = "plain")), 
           ggarrange(plot4,  ncol=1, nrow=1),
           ggarrange(plot5,  ncol=1, nrow=1),
           nrow=4, heights=c(2,1,1,1.15))
```

```
sessionInfo()
```

```
## R version 3.4.3 (2017-11-30)
## Platform: x86_64-pc-linux-gnu (64-bit)
## Running under: Ubuntu 16.10
## 
## Matrix products: default
## BLAS: /usr/lib/atlas-base/atlas/libblas.so.3.0
## LAPACK: /usr/lib/atlas-base/atlas/liblapack.so.3.0
## 
## locale:
##  [1] LC_CTYPE=en_US.UTF-8       LC_NUMERIC=C              
##  [3] LC_TIME=en_US.UTF-8        LC_COLLATE=en_US.UTF-8    
##  [5] LC_MONETARY=en_US.UTF-8    LC_MESSAGES=en_US.UTF-8   
##  [7] LC_PAPER=en_US.UTF-8       LC_NAME=C                 
##  [9] LC_ADDRESS=C               LC_TELEPHONE=C            
## [11] LC_MEASUREMENT=en_US.UTF-8 LC_IDENTIFICATION=C       
## 
## attached base packages:
## [1] stats4    parallel  stats     graphics  grDevices utils     datasets 
## [8] methods   base     
## 
## other attached packages:
##  [1] RColorBrewer_1.1-2   biomaRt_2.34.2       kableExtra_0.7.0    
##  [4] scales_0.5.0         ggpubr_0.1.6         magrittr_1.5        
##  [7] ggplot2_2.2.1        gplots_3.0.1         stringr_1.2.0       
## [10] Rsamtools_1.30.0     Biostrings_2.46.0    XVector_0.18.0      
## [13] GenomicRanges_1.30.1 GenomeInfoDb_1.14.0  IRanges_2.12.0      
## [16] S4Vectors_0.16.0     BiocGenerics_0.24.0  knitr_1.19          
## 
## loaded via a namespace (and not attached):
##  [1] Rcpp_0.12.15           prettyunits_1.0.2      gtools_3.5.0          
##  [4] assertthat_0.2.0       rprojroot_1.3-2        digest_0.6.15         
##  [7] R6_2.2.2               plyr_1.8.4             backports_1.1.2       
## [10] RSQLite_2.0            evaluate_0.10.1        highr_0.6             
## [13] httr_1.3.1             pillar_1.1.0           zlibbioc_1.24.0       
## [16] rlang_0.1.6            progress_1.1.2         curl_3.1              
## [19] lazyeval_0.2.1         gdata_2.18.0           blob_1.1.0            
## [22] rmarkdown_1.8          labeling_0.3           BiocParallel_1.12.0   
## [25] readr_1.1.1            RCurl_1.95-4.10        bit_1.1-12            
## [28] munsell_0.4.3          compiler_3.4.3         pkgconfig_2.0.1       
## [31] htmltools_0.3.6        tibble_1.4.2           GenomeInfoDbData_1.0.0
## [34] XML_3.98-1.9           viridisLite_0.3.0      dplyr_0.7.4           
## [37] bitops_1.0-6           grid_3.4.3             gtable_0.2.0          
## [40] DBI_0.7                KernSmooth_2.23-15     stringi_1.1.6         
## [43] bindrcpp_0.2           xml2_1.2.0             cowplot_0.9.2         
## [46] tools_3.4.3            bit64_0.9-7            Biobase_2.38.0        
## [49] glue_1.2.0             purrr_0.2.4            hms_0.4.1             
## [52] yaml_2.1.16            AnnotationDbi_1.40.0   colorspace_1.3-2      
## [55] caTools_1.17.1         rvest_0.3.2            memoise_1.1.0         
## [58] bindr_0.1
```
